# Anaerobic Degradation of Bicyclic Monoterpenes in *Castellaniella defragrans*

**DOI:** 10.3390/metabo8010012

**Published:** 2018-02-07

**Authors:** Edinson Puentes-Cala, Manuel Liebeke, Stephanie Markert, Jens Harder

**Affiliations:** 1 Department of Microbiology, Max Planck Institute for Marine Microbiology, Celsiusstr. 1, 28359 Bremen, Germany; epuentes@mpi-bremen.de; 2 Department of Symbiosis, Max Planck Institute for Marine Microbiology, Celsiusstr. 1, 28359 Bremen, Germany; mliebeke@mpi-bremen.de; 3 Pharmaceutical Biotechnology, University Greifswald, Felix-Hausdorff-Straße, 17489 Greifswald, Germany; stephanie.markert@uni-greifswald.de

**Keywords:** monoterpene, anaerobic metabolism, ring-opening reactions, carbon–carbon lyase, isoprenoid degradation

## Abstract

The microbial degradation pathways of bicyclic monoterpenes contain unknown enzymes for carbon–carbon cleavages. Such enzymes may also be present in the betaproteobacterium *Castellaniella defragrans*, a model organism to study the anaerobic monoterpene degradation. In this study, a deletion mutant strain missing the first enzyme of the monocyclic monoterpene pathway transformed cometabolically the bicyclics sabinene, 3-carene and α-pinene into several monocyclic monoterpenes and traces of cyclic monoterpene alcohols. Proteomes of cells grown on bicyclic monoterpenes resembled the proteomes of cells grown on monocyclic monoterpenes. Many transposon mutants unable to grow on bicyclic monoterpenes contained inactivated genes of the monocyclic monoterpene pathway. These observations suggest that the monocyclic degradation pathway is used to metabolize bicyclic monoterpenes. The initial step in the degradation is a decyclization (ring-opening) reaction yielding monocyclic monoterpenes, which can be considered as a reverse reaction of the olefin cyclization of polyenes.

## 1. Introduction

Monoterpenes are a diverse family of biogenic (C10) hydrocarbons. They are synthesized by the condensation of two activated isoprene (C5) units [1,2], primarily as secondary metabolites in higher plants. In addition, some bacteria, fungi, algae and animals are minor monoterpene producers [3]. Global estimates for monoterpene emissions into the atmosphere range from 95 to 157 Tg C a^−1^, a considerable contribution to the total biogenic organic volatiles (760–1000 Tg C a^−1^) [4,5,6,7]. Monoterpenes contained in foliage are transported into soils, sediments and aquatic habitats, where they become growth substrates for microorganisms [8,9]. Most studies on monoterpene biodegradation since the first report in 1960 were focused on aerobic microorganisms [10,11,12]. The anaerobic degradation of these biogenic hydrocarbons was discovered in the mid-1990s in denitrifying strains [13], later described as *Castellaniella* (ex *Alcaligenes*) *defragrans*, *Thauera linaloolentis* and *Thauera terpenica* [14,15,16].

*C. defragrans* metabolizes more than a dozen structurally diverse monoterpenes to carbon dioxide [15], while most studied aerobic bacteria metabolize one or two closely related substrates [17]. For this reason, over the years, *C. defragrans* (particularly strain 65Phen) has become a model organism to study the anaerobic mineralization of monoterpenes. Several enzymatic steps of the degradation of acyclic and monocyclic monoterpenes have been characterized [18,19,20,21]. The hydration of β-myrcene to (*S*)-linalool, an oxygen-independent reaction catalyzed by the linalool dehydratase/isomerase, has raised interest for its industrial potential in the production of alkenes [18,22,23]. Another novelty is the anaerobic hydroxylation of limonene to perillyl alcohol, the first step in the oxidation of monocyclic monoterpenes [19,20]. A 70 kb genetic island in *C. defragrans*’ genome seems to contain all genes necessary for the biotransformation of several monoterpenes including myrcene, linalool, geraniol, α-phellandrene, limonene and pinene to central metabolites, e.g., of the citrate cycle [19,21].

In contrast to acyclic and monocyclic monoterpenes, the enzymology of bicyclic monoterpene degradation in *C. defragrans* has not been investigated. Traces of monoterpenes were detected during growth on bicycles in the past, but a systematic study has not yet been conducted. Here, we studied the transformation of the bicyclic monoterpenes α-pinene, sabinene and 3-carene, using several experimental approaches including the metabolite accumulation in mutants with a defect in the degradation pathway for monocyclic monoterpenes.

## 2. Results

### 2.1. Metabolite Formation in Cultures

In order to identify possible intermediates in the metabolism of the bicyclic monoterpenes, cultures of *C. defragrans* strains 65Phen (wild-type, rifampicin-resistant) and 65Phen Δ*ctmAB* were grown in liquid artificial freshwater (AFW) medium with 20 mM acetate and 3 mM of either sabinene, 3-carene or α-pinene as carbon sources and 10 mM nitrate as electron acceptor. Under these conditions, the limitation of nitrate may allow the accumulation of metabolites. Cultures without inoculum or with acetate as substrate served as control experiments. Cultures were harvested in early stationary phase, and hydrophobic substances were extracted and analyzed by gas chromatography with flame ionization detection (GC-FID) for quantification and with mass spectrum detection (GC-MS) for identification. Bicyclic monoterpenes were consumed by both strains, wild-type and Δ*ctmAB* (Table 1). Sabinene concentrations were reduced to half, while only a small portion of 3-carene or α-pinene was consumed.

**Figure 1:**
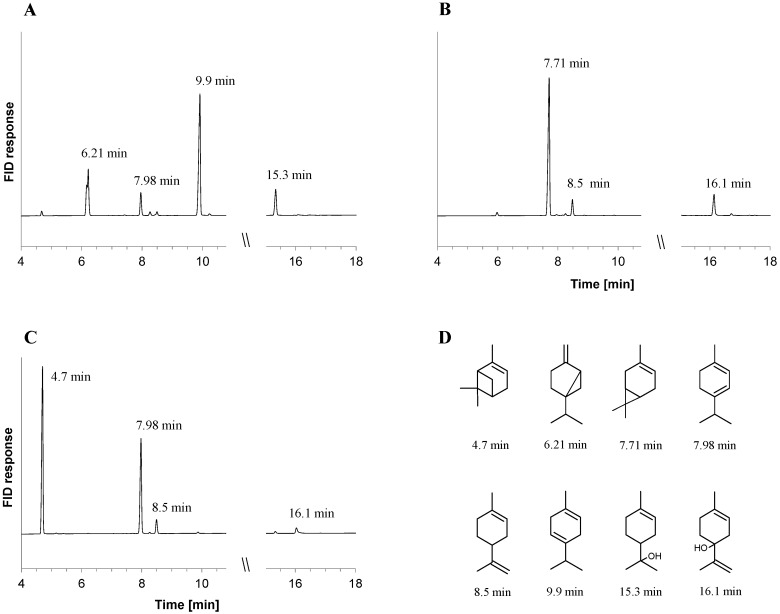
Metabolites formed in cultures of *C. defragrans* 65Phen Δ*ctmAB* grown on acetate in co-metabolism with sabinene (**A**), 3-carene (**B**) and α-pinene (**C**). Monoterpenes were identified by retention time and mass spectrum. The retention times of monoterpenes (**D**) are: α-pinene (4.7 min), sabinene (6.21 min), 3-carene (7.71 min), α-terpinene (7.98 min), limonene (8.5 min), γ-terpinene (9.9 min), terpinen-4-ol (15.3 min) and α-terpineol (16.1 min).

The decrease of bicyclic monoterpenes coincided with the formation of monocyclic monoterpenes and monoterpene alcohols in the cultures (Figure 1). These compounds were identified by retention time comparison with authentic standards and GC-MS analysis. Although both strains accumulated the same metabolites, their concentrations in wild-type cultures were considerably lower (in no case higher than 115 µM) than in cultures of the deletion mutant. The co-metabolism of acetate and sabinene in Δ*ctmAB* cultures led to the accumulation of the monoterpenes γ-terpinene (368 ± 35 µM) and α-terpinene (57 ± 7 µM), and of the monoterpene alcohol terpinen-4-ol (78 ± 11 µM) (Figure 1A). Cultures consuming 3-carene accumulated 37 ± 8 µM of limonene and 23 ± 14 µM of α-terpineol (Figure 1B). Coinciding with the disappearance of α-pinene, 227 ± 39 µM of α-terpinene, 32 ± 10 µM of limonene and 18 ± 9 µM of α-terpineol were detected in Δ*ctmAB* cultures (Figure 1C).

### 2.2. Metabolite Formation in Cell Lysates

**Figure 2:**
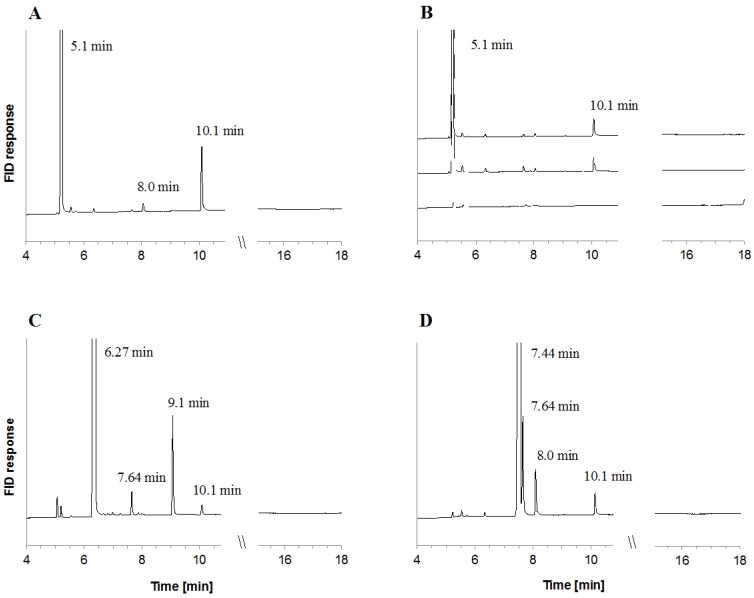
Metabolites formed in vitro from α-pinene (**A**,**B**), sabinene (**C**) and 3-carene (**D**) by dialyzed cell lysates of pinene-grown *C. defragrans* 65Phen. The *y*-axis has the same scale in all graphs; (**B**) terpinolene formation from α-pinene was hampered after EDTA addition (25 mM) (solid line) or in the absence of divalent cations and ATP (dashed line); the cell lysate did not contain monoterpenes (dotted line). Monoterpenes were identified by GC-MS, standard addition analysis and retention time comparison with authentic standards: α-pinene (5.1 min), sabinene (6.27 min), 3-carene (7.44 min) α-terpinene (7.64 min), limonene (8.0 min), γ-terpinene (9.1 min) and terpinolene (10.1 min).

The in vitro biotransformation of bicyclic monoterpenes was assayed in cell lysates of *C. defragrans* 65Phen grown on α-pinene. Several monocyclic products in the presence of each of the three bicycles as detected in hexane extracts by GC-FID (Figure 2). The main product of α-pinene isomerization was terpinolene. The formation of this product was slightly stimulated by the addition of Mg^2+^, Mn^2+^ and ATP, from 2066 to 2334 fkat mg^−1^. After removal of endogenous low-molecular weight compounds by dialysis of the cell lysate, terpinolene formation decreased and, in addition, a limonene formation appeared. The apparent enzyme activity for terpinolene synthesis increased from 957 to 2508 fkat mg^−1^ and for limonene from 110 to 196 fkat mg^−1^ after addition of the cofactors. EDTA counteracted this stimulation suggesting an essential role of the divalent ions (Figure 2B). Separate assays showed that Ca^2+^ is also a suitable enzyme cofactor (data not shown). The isomerization of sabinene and 3-carene was observed in crude and dialyzed cell lysates and also stimulated by the cofactor mixture. Incubations with sabinene yielded γ-terpinene as main product (3863 fkat mg^−1^) followed by α-terpinene (702 fkat mg^−1^) and terpinolene (445 fkat mg^−1^) (Figure 2C). The decyclization of 3-carene yielded α-terpinene (1146 fkat mg^−1^), limonene (1162 fkat mg^−1^) and terpinolene (476 fkat mg^−1^) (Figure 2D). The ring-opening of the bicyclic monoterpenes was observed in cell lysates, but not in the soluble or the membrane fraction obtained after separation by ultracentrifugation (230,000× *g* for 40 min at 4 °C). The recombination of the resuspended 75 Svedberg membrane pellet with the supernatant did not restore enzyme activity. This suggests an irreversible disruption of an enzyme complex and may be considered in future attempts to purify the enzyme.

### 2.3. Differential Proteomics

Growth of wild-type *C. defragrans* in α-phellandrene resulted in the upregulated content of 107 proteins in comparison to the proteome of acetate-grown cells [19]. In this study, we investigated the proteomes of *C. defragrans* 65Phen grown on acetate or on one of the monoterpenes R-(+)-limonene, sabinene and α-pinene as single carbon source to identify links between the metabolism of bicyclic and monocyclic monoterpenes. Soluble proteins were separated by anion exchange chromatography and one-dimensional denaturing gel electrophoresis and then protein bands were identified by MALDI-ToF-MS. This approach detected 33 proteins with higher concentration in monoterpene-grown cells (Table 2).

Twenty-seven of the identified proteins were present in larger concentrations in all three monoterpene substrates. Among them were several proteins encoded in the genomic island for monoterpene degradation and known to participate in the metabolism of monocyclic monoterpenes. Proteins CtmA, CtmB and CtmF (CDM25290, CDM25289 and CMD25285, respectively) are synthesized from the gene cluster *ctmABCDEFG*, known to be responsible for the hydroxylation of limonene to perillyl alcohol [19,21]. GeoA (CDM25267) and GeoB (CDM25281) catalyze the oxidation of perillyl alcohol and perillyl aldehyde, respectively, resulting in the formation of perillic acid [20]. These and another eight proteins (CDM23572, CDM24415, CDM25009, CDM25241, CDM25246, CDM25251, CDM25259 and CDM25260) were previously detected as proteins with a higher cellular concentration during growth of *C. defragrans* on the monocyclic monoterpene α-phellandrene [19]. These findings link the pathway for bicyclic monoterpene degradation with that for monocyclic monoterpenes. Still, these experiments did not identify a candidate protein responsible for the cycloisomerization of the bicyclic substrates.

### 2.4. Transposon Conjugants Affected Growth on Monoterpenes

To discover the genes involved in bicyclic monoterpene metabolism, random transposon mutagenesis was performed by means of the mini-Tn5 transposon hosted in the vector pRL27 [24]. Delivery into *C. defragrans* 65Phen by biparental conjugation resulted in the generation 8896 transconjugants growing with acetate on solid media. These strains were screened on solid medium replacing acetate by one of the monoterpenes limonene, sabinene, α-pinene, or 3-carene supplied via the gas phase. Forty-two of the transconjugants showed impaired growth on at least one of the monoterpenes. Thirty-five unique transposon insertion sites were identified by bidirectional sequencing after rescue cloning (Table 3). The mutation rate of 3.93 × 10^−3^ was in the range previously observed in similar transposon studies [19,24,25]. Three transposon insertions occurred within the genetic island for monoterpene degradation affecting proteins CDM25252, CDM25285 and CDM25290 (genes *mrcD*, *ctmF* and upstream of *ctmA*, respectively) [19]. Insertions in other genes involved in β-oxidation-like reactions, amino acid degradation and the methylisocitrate cycle (CDM25923, CDM22783 and CDM25080, respectively) also resulted in low to no bacterial growth on monoterpenes (Table 3).

Four ABC transporter genes were inactivated in six transconjugants. The corresponding proteins CDM23032 and CDM23105 had been annotated as components of nutrient uptake systems, whereas CDM23018 and CDM24629 were associated to the export of toxins and antibiotics. Inactivated was also a fifth transporter (CDM24678) that affiliated to the superfamily of proton-driven drug/metabolite transporters. Other transposon insertions took place in genes related to miscellaneous amino acid metabolism (CDM22986, CDM24922), cell wall synthesis (CDM24591), water homeostasis (CDM23452, CDM24706) and several other genes, which currently cannot be linked to monoterpene metabolism, but may contribute to the integrity of the cell in the presence of toxic monoterpenes. Remarkably, several transposon insertions occurred in non-coding regions in front of genes. Seven out of 11 of these insertions were more than 250 bp upstream of the start codon. For the remaining four mutants (CDM22986, CDM23110, CDM25290 and CDM25994), the insertion occurred less than 100 bp upstream of the respective transcription start sites, which might have affected gene expression.

## 3. Discussion

In the past, traces of α-terpinene, cymene and limonene (4–20 µM) were observed in cultures of *C. defragrans* grown with bicyclic monoterpenes [13]. Here, we elevated the concentrations of intermediate metabolites using a mutant strain unable to mineralize monocyclic monoterpenes and a cometabolic metabolism with acetate as growth substrate. The mutant strain formed larger amounts than the wild type strain, but the pattern of the monoterpene metabolites remained stable. We observed the formation and accumulation of several monocyclic monoterpenes (18–368 µM), which all support as individual substance growth of *C. defragrans* [15]. These observations indicate that the traces of monoterpenes found in wild-type cultures indeed are not accidental side-products, but true intermediates of the degradation pathways.

**Figure 3:**
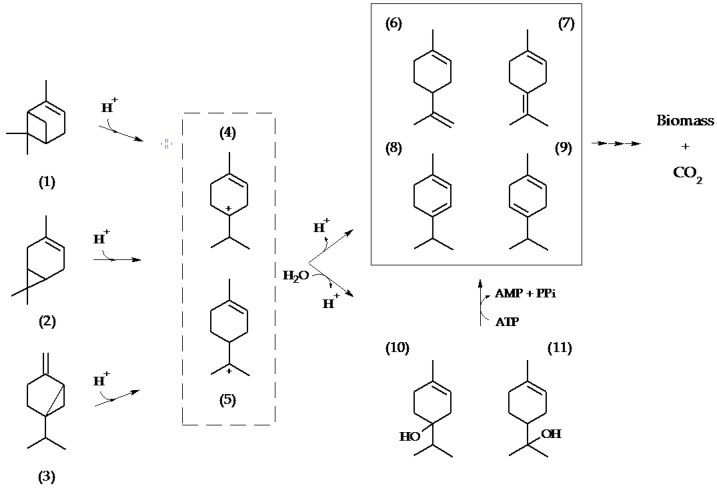
Proposed pathway for the degradation of the bicyclic monoterpenes α-pinene (1), 3-carene (2) and sabinene (3) in *C. defragrans* 65Phen. Electrophilic attack on the allylic double bond and an intramolecular rearrangement generates the monocyclic transient carbocations terpinen-4-yl (4) and α-terpinyl (5). These can deprotonate into monocyclic monoterpene olefins such as (6), (7), (8), (9) or hydroxylated to (10) and (11) by water addition. The alcohols may be dehydrated via an ATP-dependent reaction to monoterpene olefins.

The isomerization of the bicyclic substrates into monocyclic monoterpenes requires the opening of the cyclopropyl ring present in carene and sabinene as well as the cyclobutyl ring in pinene. This process is expected to involve a transient carbocation intermediate [26,27]. In 1968, Shukla and Bhattacharyya proposed a carbocation as precursor for all monocyclic products of an aerobic *Pseudomonas* strain metabolizing α- and β-pinene [28], a hypothesis that has since gained additional evidence [26,29,30]. The products detected in *C. defragrans* suggest the transient formation of terpinen-4-yl and α-terpinyl carbocations, yielding either monocyclic olefins via deprotonation (limonene, terpinolene) or monoterpene alcohols via combination with hydroxide ions or water. The stimulating role of divalent cations and ATP in the synthesis of unsubtituted monocyclic monoterpenes is an aim for future research, but may involve enzyme modifications or the dehydroxylation of these alcohol intermediates via phosphorylation and elimination of phosphate or pyrophosphate (Figure 3). 

The detection of several proteins involved in monocyclic monoterpene degradation provided additional indirect evidence for the mineralization of bicyclic monoterpenes via monocyclic intermediates. In addition, insertion mutations affecting growth on bicyclic monoterpenes were identified in genes (or upstream of genes) required for the mineralization of monocyclic monoterpenes. Transposon mutants were also affected in genes predicted to be part of defense or detoxification mechanisms such as outer membrane components or membrane permeases, linking the phenotypes to the toxicity of monoterpenes [19,25]. In summary, our observations suggest an isomerization of bicyclic monoterpenes into monocyclic intermediates, prior to their complete mineralization via the monocyclic monoterpene pathway [19]. These decyclization reactions were stimulated by divalent ions and ATP, but a detailed understanding has to await the isolation of the enzyme. So far, the involvement of α-terpinyl and terpinen-4-yl carbocations does best explain the formation of monocyclic monoterpenes.

## 4. Methods

### 4.1. Bacterial Cells 

The strains and plasmids used in this study are listed in Table 4.

The in-frame deletion mutant Δ*ctmAB* was prepared as described elsewhere [31] and kindly provided by Jan Petasch (Max Planck Institute for Marine Microbiology, Bremen, Germany).

### 4.2. Culture Conditions 

Liquid cultures of *Castellaniella defragrans* 65Phen and all mutants thereof grew under anoxic denitrifying conditions in Artificial Fresh Water (AFW) medium as described [19]. Unless otherwise indicated, 20 mM of sodium acetate or 3 mM of monoterpene was provided as carbon sources. Monoterpenes were supplied in liquid cultures by means of the carrier phase 2,2,4,4,6,8,8-heptamethylnonane (HMN). Cultures were incubated at 28 °C under constant shaking (60 rpm). *E. coli* BW20767 was grown at 37 °C under constant shaking (140 rpm) in Luria Bertani (LB) broth. The plasmid pRL27 was maintained in cultures containing 25 µg mL^−1^ of kanamycin.

### 4.3. Metabolite Analysis

*C. defragrans* strains 65Phen and Δ*ctmAB* were inoculated in 10 ml liquid AFW medium containing 20 mM acetate and 3 mM of sabinene, 3-carene or α-pinene. The monoterpenes were dissolved in a filter-sterilized 10% *v*/*v* Tween 20 solution. Tween 20 was not a growth substrate and had a final concentration of 0.5% *v*/*v* in the medium. After 7 days of culture growth, the metabolites were salted out from the aqueous phase by addition of 500 µL of isopropanol, followed by the addition of 5 g of K_2_CO_3_. The suspension was homogenized for 20 min at 60 rpm for and then stood vertically 10 min for phase separation. One µL of the upper (organic) phase was analyzed in a gas chromatograph (Perkin Elmer Auto System XL, Überlingen, Germany) equipped with an Optima^®^-5 column (0.25-μm film thickness, 50 m × 0.32-mm inner diameter; Macherey-Nagel, Düren, Germany) and coupled to a flame ionization detector (FID). The temperature program was: injection port 250 °C; column for 2 min at 40 °C, increate at a rate of 4 °C min^−1^ until 100 °C, constant for 0.1 min, at a rate of 45 °C min^−1^ up to 320 °C, and constant at 320 °C for 3 min; detection temperature 350 °C. The split ratio was 1:9. FID responses for R-(+)-limonene, α- and γ-terpinene, terpinolene and α-terpineol were 5.26 ± 0.24 mV*s per picomol carbon. For terpinen-4-ol, the response was 4.1 mV*s per picomol carbon. Calibration curves for monoterpene dienes were linear within the range of 0.1–10 nmol of injected monoterpene. For terpinen-4-ol and α-terpineol, linearity ranged between 0.5 and 10 nmol. In all cases, the coefficient of determination (R^2^) was higher than 0.99. Monoterpene concentrations refer to their theoretical concentrations in the aqueous phase. Metabolite identity initially defined by retention time was confirmed by GC-MS analysis on an Agilent 7890B gas chromatograph (Agilent, Santa Clara, CA, USA) connected to an Agilent 5977A Mass-selective detector. Analyte separation was performed on an Agilent 30 m DB5-MS column with a 10 m DuraGuard column applying the following temperature program: injection port temperature at 250 °C, initial column temperature 40 °C for 1 min, increasing to 160 °C at 4 °C min^−1^ further to 280 °C at 30 °C min^−1^ and hold for 1 min. For verification, mass spectra of monoterpene standards were recorded as reference. Metabolites were then identified by their mass spectra. 

### 4.4. Enzyme Assays

All activity experiments were performed in the absence of oxygen. Cells of wild-type *C. defragrans* grown anaerobically on α-pinene were resuspended in 25 mM Tris-Cl, pH 8.0 and disrupted by two passages through a French-Press cell disrupter (SLM Aminco, Rochester, NY, USA) at 8.6 MPa. The crude cell lysate, as well as the soluble and pelleted fractions obtained after ultracentrifugation (230,000× *g* for 30 min at 4 °C), were dialyzed in anoxic 25 mM Tris-Cl, pH 8.0 (1:10^6^) and tested for enzyme activity. A 500 µL assay contained 2.5–5 mg protein, 2 mM dithiothreitol, 5 mM Mg^2+^, 10 mM Mn^2+^, 10 mM ATP, 0.5% Tween 20 and 60 mM of a bicyclic monoterpene. When indicated, divalent cations were replaced for 10 mM Ca^2+^ supplied as CaCl_2_. The reactions were initiated by monoterpene addition and incubated for 16–18 h at 28 °C. Hydrophobic metabolites were extracted with 100 µL *n*-hexane and analyzed by GC-FID as aforementioned. Retention times shown in Figure 1 and Figure 2 are shifted due to column shortening during equipment maintenance. Monoterpene identity was confirmed by analysis with internal standards and GC-MS analysis.

### 4.5. Differential Proteomics: Preparation of Cell Lysates, Soluble Protein Fractionation and MALDI-ToF Analysis

Cells of *C. defragrans* were cultivated in 10 mL of AFW medium supplemented with either 20 mM acetate or 3 mM of R-(+)-limonene, 3-carene, sabinene or α-pinene and sodium nitrate (10 mM). Growth was determined as increase in optical density at 600 nm. Transfers to fresh medium were carried out once a week with an inoculum of 2% vol/vol. After at least 5 consecutive transfers, 5 mL of 5 to 7 days-grown cultures were transferred into 2000 mL of fresh AFW. At this scale, nitrate concentration was increased to 20 mM and the monoterpenes were added without a carrier phase. The biomass was harvested at late exponential phase and resuspended in 10 mM potassium phosphate, pH 8.0. After cell disruption, soluble proteins were separated from cell debris by ultracentrifugation at 230,000× *g* for 40 min at 4 °C. Protein concentration was determined by the method of Bradford [33] with bovine serum albumin as the standard. 20 to 40 mg of the soluble proteins were loaded onto a Resource^TM^ Q column (GE Healthcare, Freiburg, Germany) with a 1 mL bed volume installed in an ÄKTA purifier system (GE Healthcare, Freiburg, Germany) that was equilibrated with 10 mM potassium phosphate, pH 8.0. Protein fractionation was performed by applying a salinity gradient from 0–500 mM KCl over 50 nominal column volumes (flow rate: 0.5 mL min^−1^) with a collection in 25 fractions of 2 mL. Unbound proteins (flow-through) were collected as a single additional fraction. Each fraction was separated by size and stained in one-dimensional SDS-PAGE as described by Laemmli [34]. A comparison identified proteins present in different amounts. These were excised, subjected to tryptic in-gel digestion and analyzed on an AB SCIEX TOF/TOF^TM^ 5800 Analyzer (AB Sciex, Darmstadt, Germany).

### 4.6. Transposon Mutagenesis

The generation of transposon insertion mutants and the evaluation of their ability to grow on monoterpenes proceeded as described by Petasch et al. [19]. Mutants with low to no growth on limonene, 3-carene, sabinene and/or α-pinene were selected for further examination. The genomic localization of the Tn5 mini-transposon in each mutant was determined by sequencing after rescue cloning. DNA was extracted from mutant cells from over-night cultures grown in LB medium [35]. One µg of genomic DNA was digested with BamHI (Thermo Fisher Scientific, Waltham, MA, USA) and purified with the GeneJET PCR Purification Kit (Thermo Fisher Scientific). The BamHI fragments were circularized with T4 DNA ligase (Thermo Fisher Scientific). The circularized DNA fragments were transformed into chemically competent *E. coli* BW20767 (*pir*^+^). Clones with the ability to grow on kanamycin-containing LB medium were selected and plasmids were extracted (QIAprep Spin Miniprep Kit, QIAGEN, Hilden, Germany). Sequencing reactions were prepared with 100 to 200 ng of plasmid DNA and the BigDye Terminator v3.1 Cycle Sequencing Kit (Applied Biosystems, Life Technologies Corporation, Carlsbad, CA, USA). The oligonucleotides tpnRL17-1 (AACAAGCCAGGGATGTAA) and tpnRL 13-2 (CAGCAACACCTTCTTCACGA) were used to prime the reactions [24]. The temperature program used was 96 °C for 5 min, 99 cycles of 96 °C for 20 s, 55 °C for 10 s and 60 °C for 5 min. The DNA sequences were read on an ABI Prism 3130*xl* Genetic Analyzer (Applied Biosystems Life Technologies Corporation, Carlsbad, CA, USA).

## Figures and Tables

**Table 1:** Recovery of bicyclic monoterpenes from cultures of *C. defragrans* wild-type and Δ*ctmAB.*

Monoterpene Co-Substrate	Monoterpene (mM), (Monoterpene Consumed (%))	
	Wild-Type	Δ*ctmAB*
sabinene	1.6 ± 0.6 (47%)	1.44 ± 0.11 (52%)
3-carene	2.65 ± 0.15 (12%)	2.68 ± 0.02 (11%)
α-pinene	2.95 ± 0.03 (2%)	2.69 ± 0.06 (10%)

**Table 2:** Proteins of *C. defragrans* 65Phen present in larger amounts in cells grown on monoterpenes compared to cells grown on acetate. Proteins were fractionated with anion exchange chromatography, semi-quantified by SDS-PAGE (In-gel protein band intensity: +, observable; (+) weakly observable; -, not visible) and identified by MALDI-ToF-MS. Carbon source: L, R-(+)-limonene (monocyclic); S, sabinene (bicyclic); P, α-pinene (bicyclic).

Accession Number	Annotation	Growth Substrate		
		L	S	P
CDM22609	Translation elongation factor Tu	+	-	-
CDM22610	Translation elongation factor G	+	+	+
CDM22641	Translation elongation factor Tu	+	-	-
CDM22907	Copper-containing nitrite reductase	+	+	+
CDM23001	Aconitate hydratase	+	+	+
CDM23572	Methylmalonate-semialdehyde dehydrogenase	+	+	+
CDM23679	Heat shock protein 60 family chaperone GroEL	+	+	+
CDM23795	Chaperone protein DnaK	+	+	+
CDM23915	3-methylmercaptopropionyl-CoA dehydrogenase DmdC	+	+	+
CDM24415	Hypothetical protein	+	+	+
CDM24550	Glutamine synthetase type I	+	+	+
CDM24733	Isoleucyl-tRNA synthetase	+	+	+
CDM24892	Membrane alanine aminopeptidase N	-	+	+
CDM24998	Transcription termination protein NusA	+	-	+
CDM25009	Acetoacetyl-CoA reductase	+	+	+
CDM25072	Citrate synthase	+	+	+
CDM25085	Aconitate hydratase 2	+	+	+
CDM25210	Polyribonucleotide nucleotidyltransferase	(+)	+	+
CDM25241	Acyl-CoA dehydrogenase	+	+	+
CDM25246	3-hydroxyacyl-CoA dehydrogenase	(+)	+	+
CDM25251	4-isopropenyl-2-oxo-cyclohexane-1-carboxyl-CoA hydrolase MrcE	(+)	+	+
CDM25253	2,4-enoyl-CoA reductase MrcC	+	+	+
CDM25259	RND efflux transporter component	+	+	+
CDM25260	RND efflux transporter component	+	+	+
CDM25267	Geraniol dehydrogenase GeoA	(+)	+	+
CDM25281	Geranial dehydrogenase GeoB	-	+	+
CDM25285	NADH:ferredoxin oxidoreductase CtmF	+	+	+
CDM25289	Limonene dehydrogenase CtmB	+	+	+
CDM25290	Limonene dehydrogenase CtmA	+	+	+
CDM25340	Branched-chain amino acid aminotransferase	-	+	+
CDM25770	Protein export cytoplasm protein SecA	-	-	+
CDM25844	Heat shock protein 60 family chaperone GroEL	+	+	+
CDM26013	Glutamate aspartate periplasmic binding protein precursor GltI	+	+	+

**Table 3:** Genetic and physiological features of *C. defragrans* 65Phen transconjugants affected in the metabolism of monoterpenes. Growth on monoterpenes was tested on solid and in liquid media. Carbon sources: L, R-(+)-limonene; S, sabinene; P, α-pinene; C, 3-carene. Growth quantification in liquid was based on ΔOD_600_: + = ΔOD_600_ ≥ 0.15; (+) ≥ 0.05; - < 0.05.

Affected Protein (acc. no.)	Gen Length [bp]	Insertion Position [bp]	Annotation	Growth on Solid Media				Growth in Liquid Media			
				L	S	P	C	L	S	P	C
Wild-Type	-	-	-	+	+	+	+	+	+	+	+
CDM22783	1217	1023	Cystathionine beta-lyase	-	-	-	-	-	-	-	-
CDM23032	702	793	Glutamate ABC transporter permease (periplasmic component)	-	-	-	-	+	-	-	-
CDM23105	930	627	High-affinity branched-chain amino acid transport system (permease protein) LivH	-	-	-	-	-	-	-	-
CDM23105	930	161	High-affinity branched-chain amino acid transport system (permease protein) LivH	(+)	(+)	-	-	+	-	-	-
CDM23279	2793	411	Inositol phosphate phosphatase	(+)	-	-	-	-	(+)	(+)	-
CDM23452	1230	150	Fused spore maturation proteins A and B	(+)	-	-	(+)	(+)	(+)	-	(+)
CDM23960	1230	765	Glycolate dehydrogenase (iron-sulfur subunit)	-	-	-	-	+	-	-	(+)
CDM24063	279	243	HigB toxin protein	-	-	-	(+)	(+)	-	-	(+)
CDM24591	3630	889	*O*-antigen biosynthesis protein	(+)	-	-	-	(+)	-	-	-
CDM24600	1029	1012	Glycosyl transferase, family 2	-	-	-	(+)	+	+	-	-
CDM24629	1116	11	ABC transport system (permease component) YbhR	-	-	-	-	-	-	-	(+)
CDM24629	1116	1104	ABC transport system (permease component) YbhR	-	-	-	-	-	-	-	-
CDM24678	897	388	Permease of the drug/metabolite transporter superfamily	-	-	(+)	(+)	-	-	(+)	(+)
CDM24706	3387	2695	Trehalose synthase	-	-	-	-	-	-	-	-
CDM24919	3840	1904	Putative ATP-dependent helicase	-	-	-	-	-	-	-	-
CDM24922	1356	1253	*N*-acetylglutamate synthase	-	-	(+)	(+)	(+)	+	-	-
CDM25080	900	88	Methylisocitrate lyase	(+)	-	-	-	(+)	+	+	-
CDM25154	525	334	Hypothetical protein	(+)	-	-	-	+	+	-	+
CDM25252	771	497	2-hydroxy-4-isopropenyl-cyclohexane-1-carboxyl-CoA dehydrogenase MrcD	(+)	-	-	-	-	-	-	-
CDM25285	1230	492	NADH:ferredoxin oxidoreductase CtmF	-	-	-	-	-	-	-	-
CDM25322	999	468	Hypothetical protein	-	(+)	-	-	+	(+)	(+)	-
CDM25752	849	330	Glucose-1-phosphate thymidylyltransferase	(+)	+	-	-	+	+	-	-
CDM25923	777	223	Enoyl-CoA hydratase	-	-	-	-	-	-	-	-
CDM26084	1514	1428	Putative transposase	-	-	-	(+)	(+)	-	(+)	+
Non-coding region	-	−500	Upstream of CDM22657: single-stranded DNA-binding protein	-	-	-	(+)	+	+	(+)	-
Non-coding region	-	−10	Upstream of CDM22986: phenylacetate-CoA ligase	(+)	-	-	-	+	+	-	-
Non-coding region	-	26	Downstream of CDM23018: auxin efflux transporter	-	-	-	(+)	-	-	-	+
Non-coding region	-	−387	Upstream of CDM23059: hypothetical protein	-	-	-	-	-	-	-	-
Non-coding region	-	−97	Upstream of CDM23110: glutamate ABC transporter (periplasmic component)	-	-	-	-	+	-	-	-
Non-coding region	-	−281	Upstream of CDM23992: hypothetical transcriptional regulator	-	(+)	-	-	(+)	(+)	-	-
Non-coding region	-	−559	Upstream of CDM23992: putative transposase	(+)	-	-	(+)	+	-	-	-
Non-coding region	-	−270	Upstream of CDM23993: putative transcriptional regulator	(+)	-	-	-	+	+	-	-
Non-coding region	-	−78	Usptream of CDM25290: limonene dehydrogenase CtmA	(+)	(+)	(+)	-	-	(+)	-	-
Non-coding region	-	−40	Upstream of CDM25994: thiamin-phosphate pyrophosphatase	-	-	-	-	-	-	-	-
Non-coding region	-	−295	Upstream of CDM26087: putative transposase	(+)	-	-	-	+	-	-	-

**Table 4:** List of bacterial strains used in this study.

Strain	Genotype	Source
*C. defragrans* 65Phen	Rifampicin-resistant (Ra^R^)	[31]
*C. defragrans* 65Phen Δ*ctmAB*	65Phen, Ra^R^, Δ*ctmAB*	This work
*E. coli* BW20767	RP4-2-*tet*::Mu-1, *kan*::Tn7 integrant, *leu-63*::*IS10*, *recA1*, *creC510*, *hsdR17*, *endA1*, *zbf-5*, *uidA*, (Δ*Mlul*)::*pir*^+^ *thi*	[32]
Plasmid		
pRL27	Tn5 with Km^R^, R6K *ori*, *oriT*, RP4, *tnpA*	[24]
